# FOS Rescues Neuronal Differentiation of Sox2-Deleted Neural Stem Cells by Genome-Wide Regulation of Common SOX2 and AP1(FOS-JUN) Target Genes

**DOI:** 10.3390/cells10071757

**Published:** 2021-07-12

**Authors:** Miriam Pagin, Mattias Pernebrink, Mattia Pitasi, Federica Malighetti, Chew-Yee Ngan, Sergio Ottolenghi, Giulio Pavesi, Claudio Cantù, Silvia K. Nicolis

**Affiliations:** 1 Department of Biotechnology and Biosciences, University of Milano-Bicocca, Piazza della Scienza 2, 20126 Milano, Italy; miriam.pagin@unimib.it (M.P.); m.pitasi@campus.unimib.it (M.P.); f.malighetti@campus.unimib.it (F.M.); sergio.ottolenghi@unimib.it (S.O.); 2 Wallenberg Centre for Molecular Medicine, Linköping University, SE-581 83 Linköping, Sweden; mattias.pernebrink@liu.se; 3 Department of Biomedical and Clinical Sciences, Division of Molecular Medicine and Virology, Faculty of Medicine and Health Sciences, Linköping University, SE-581 83 Linköping, Sweden; 4 The Jackson Laboratory for Genomic Medicine, Farmington, CT 06032, USA; Chewyee.Ngan@jax.org; 5 Department of Biosciences, University of Milano, Via Celoria 26, 20134 Milano, Italy; giulio.pavesi@unimi.it

**Keywords:** neural stem cells, neurogenesis, gliogenesis, CUT&RUN, transcription factors, chromatin, Sox2, Fos, Socs3

## Abstract

The transcription factor SOX2 is important for brain development and for neural stem cells (NSC) maintenance. *Sox2*-deleted (Sox2-del) NSC from neonatal mouse brain are lost after few passages in culture. Two highly expressed genes, *Fos* and *Socs3*, are strongly downregulated in Sox2-del NSC; we previously showed that *Fos* or *Socs3* overexpression by lentiviral transduction fully rescues NSC’s long-term maintenance in culture. Sox2-del NSC are severely defective in neuronal production when induced to differentiate. NSC rescued by *Sox2* reintroduction correctly differentiate into neurons. Similarly, *Fos* transduction rescues normal or even increased numbers of immature neurons expressing beta-tubulinIII, but not more differentiated markers (MAP2). Additionally, many cells with both beta-tubulinIII and GFAP expression appear, indicating that FOS stimulates the initial differentiation of a “mixed” neuronal/glial progenitor. The unexpected rescue by FOS suggested that FOS, a SOX2 transcriptional target, might act on neuronal genes, together with SOX2. CUT&RUN analysis to detect genome-wide binding of SOX2, FOS, and JUN (the AP1 complex) revealed that a high proportion of genes expressed in NSC are bound by both SOX2 and AP1. Downregulated genes in Sox2-del NSC are highly enriched in genes that are also expressed in neurons, and a high proportion of the “neuronal” genes are bound by both SOX2 and AP1.

## 1. Introduction

The SOX2 transcription factor is expressed in the central nervous system (CNS) from the earliest developmental stages throughout vertebrate evolution [1,2]. Heterozygous loss-of-function mutations of *SOX2* in humans lead to a spectrum of CNS abnormalities, including vision defects, hippocampal defects, and intellectual disability, pointing to important functions in many aspects of neurodevelopment [1,2]. In mice, SOX2 functional roles in the CNS have been demonstrated by *Sox2* hypomorphic mutants as well as by conditional *Sox2* knockouts [3,4,5,6]. At the cellular level, SOX2 was found to play an essential function in the maintenance of neural stem cells (NSC) in the developing CNS and, postnatally, in both the lateral ventricle (LV) and hippocampus dentate gyrus (DG) [3,5,7]. A requirement for *Sox2* in the long-term maintenance of brain-derived NSC grown in vitro was confirmed by additional studies [3,8,9]. On the other hand, the terminal differentiation of NSC into neurons was also found to require *Sox2*. Indeed, GABAergic neurons migrating during development from the ganglionic eminence towards the cortex are abnormal in *Sox2* mutants [10]. Further, in the postnatal hippocampus, partial *Sox2* deficiency in mice that are compound heterozygous for hypomorphic and null *Sox2* alleles resulted in a decreased in the fraction of proliferating (BrdU-positive) NSC that give rise to terminally differentiated neurons [5]. Accordingly, NSC from such hypomorphic *Sox2*-mutant brains and cultured in vitro in differentiating conditions, display a specific impairment in the production of terminally differentiated neurons; this defect is rescued by lentiviral transduction of *Sox2* at early (but not late) stages of in vitro differentiation [10]. A role for SOX2 in neuronal differentiation was also documented in mice, in projection neurons of the visual thalamus, which require SOX2 for their maturation and the establishment of appropriate connectivity with the retina and cerebral cortex [11]. Finally, a neuronal differentiation function for SOX2 was suggested to occur in different species, such as C. elegans [2].

The functional involvement of SOX2 in neuronal differentiation raises the question of which downstream genes mediate this function, and of which molecular mechanisms are involved. In this work, we report that *Fos*, one of the genes most downregulated in NSC following *Sox2* neural conditional knockout [8], is able, when introduced into *Sox2*-deleted NSC via a lentiviral vector, to substantially rescue their neuronal differentiation defect. Gene ontology analysis of RNA-seq data highlights that the most represented functional category among genes downregulated in *Sox2*-deleted versus wild-type NSC is represented by genes involved in neuronal differentiation. Strikingly, CUT&RUN analysis of FOS, JUN (together forming the AP1 complex), and SOX2 in NSC chromatin reveals a broad, genome-wide co-binding of SOX2 and AP1 to neuronal differentiation genes expressed in NSC. This suggests the interesting possibility that neuronal differentiation is set in motion by a gene regulatory network jointly regulated by SOX2 and AP1.

## 2. Materials and Methods

### 2.1. Primary Ex-Vivo Neural Stem/Progenitor Cell Cultures

Brain-derived NSC cultures were obtained from dissected telencephalon of wild-type and Sox2-deleted P0 mice [3,8] and grown in fresh medium (FM): DMEM-F12 with Glutamax (GIBCO, Milan, Italy), supplemented with 1/50 *v/v* B27 (Life Technologies, Milan, Italy), 1% of penicillin-streptomycin (Euroclone, Milan, Italy) supplemented with EGF (10 ng/mL, Tebu-bio, Milan, Italy) and bFGF (10 ng/mL, Tebu-bio, Milan, Italy) as a mitogen, as described in [3,8,9,12].

### 2.2. RNA-seq

RNA-seq data for undifferentiated (differentiation day 0, D0) normal (WT), and Sox2-deleted (Sox2-del) NSC were previously reported [8]. RNA-seq analysis of the same cells (three WT, three Sox2-del populations, see Table 1) at D4 and D11 of differentiation was performed in parallel to the D0 analysis in exactly the same way as for D0. D4 and D11 RNA-seq raw data were processed, from expression quantification to differential expression analysis, following the same pipeline described in [8] for D0. Expression data, false discovery rate (FDR), and fold-change (FC) values are available in Table S1. Raw data can be found under accession number GEO: GSE90561.

### 2.3. Lentiviral Constructs

The Fos expressing lentivirus was previously described in [9]: the Fos cDNA was cloned into a unique BamHI site upstream to the IRES-dNGFR cassette of the pHR SIN BX IR/EMW [13] (provided by A. Ronchi, Milan, Italy). The Sox2 and Socs3-expressing lentiviruses were previously described [3,8,9] and also expressed the GFP fluorescent marker.

Lentiviral vectors were produced by calcium phosphate transfection into the packaging human embryonic kidney cell line 293T, of the VSV-G plasmid (encoding ENV), CMV R8.74 (packaging), and pRSV-REV (encoding reverse transcriptase) [8,9,13]. Briefly, after transfection, following replacement with DMEM high glucose (Euroclone, Milan, Italy), containing 10% fetal bovine serum (Sigma, Milan, Italy), 1% penicillin-streptomycin (Euroclone, Milan, Italy), 1% of L-glutamine (Euroclone, Milan, Italy), the cell supernatants were collected 72 h after transfection. Lentiviral vectors were titrated on HEK-293T cells by measuring the percentage of eGFP (for the Sox2 and Socs3-transducing vectors) and of dNGFR-positive cells (for the Fos-transducing vector) by flow cytometry [9,13].

### 2.4. Sox2-del Transduction with Lentiviral Constructs Encoding Sox2, Socs3 and Fos

Sox2-del NSC transduction with lentiviral constructs, expressing *Sox2*, *Fos*, or *Socs3*, was as in [8,9,14]. After 6–8 passages in NSC proliferation medium [8,9], during which essentially all cells had become positive for the virally transduced *Sox2*, or *Fos*, or *Socs3* expression construct [8,9], cells were induced to differentiate.

### 2.5. RT-PCR Analysis of Sox2, Fos, and Socs3

The RT-PCR analysis of *Sox2*, *Fos*, and *Socs3* in Figure S4 was performed as in [9]. The primers used for *Socs3* and *Fos* mRNA detection were as in [9]; primers for *Sox2* mRNA detection were:

Sox2_Fw GGCAGCTACAGCATGATGCAGGAGC;

Sox2_Rev CTGGTCATGGAGTTGTACTGCAGG.

Sox2-del NSC transduced with *Sox2*, or *Fos*, or *Socs3* expression vectors and kept in culture for more than eight passages were analyzed in parallel with WT NSC and Sox2-del NSC at early (4–6) passages.

### 2.6. NSC Differentiation

Sox2-del NSC transduced with *Sox2*, or *Fos*, or *Socs3* expression vectors were kept in long-term culture and differentiated after 6–8 passages or later, when most of the NSC appeared to harbor the transduced vector [8,9]. Sox2-del NSC were differentiated before passage 6, when they were still efficiently growing. NSC differentiation was as in [10]. Briefly, WT, Sox2-del, Sox2-del + *Sox2*, Sox2-del + Fos, and Sox2-del + *Socs3* NSC were dissociated to single cells, and plated onto MATRIGEL (Becton Dickinson, NJ, USA) coated glass coverslips at 4 *×* 10^4^ cells/1 mL/well in 24-well plates in FM with bFGF only as the mitogen [10]. After 4 days, the medium was changed to neural stem cell medium without bFGF, supplemented with 1% embryonic stem-cell FBS (Thermo Fisher, Milan, Italy). After a further seven days (differentiation day 11), cells were analyzed by immunocytochemistry.

### 2.7. Immunocytochemistry

At differentiation day 11, cells were fixed (20 min) with 4% PFA in phosphate-buffered saline (PBS; pH 7.4) and rinsed three times with PBS. Coverslips were then incubated for 90 min at 37 °C in PBS containing 10% normal goat serum (NGS), 0.2% Triton X-100. Coverslips were incubated with the primary antibodies overnight at 4 °C. Following thorough washing with PBS, cells were incubated for 45 min (room temperature) with specific secondary antibodies (1:1000, AlexaFluor Invitrogen, Milan, Italy). Coverslips were rinsed three times in PBS and mounted on glass slides with Fluoromount (Sigma, Milan, Italy) with DAPI (4′,6-diamidino-2-phenylindole).

Primary antibodies:

anti-β-TubIII (rabbit polyclonal IgG, 1:400, Covance, NJ, USA),

anti-MAP2 (mouse monoclonal IgG1, 1:100 Merck, Darmstadt, Germany),

anti-GFAP (mouse monoclonal IgG1, 1:100 Merck, Darmstadt, Germany),

Secondary antibodies:

goat anti-rabbit IgG 488 AlexaFluor (1:1000, Invitrogen, Milan, Italy),

goat anti-mouse IgG1 594 AlexaFluor (1:1000, Invitrogen, Milan, Italy).

### 2.8. GO Enrichment Analysis

Published RNA-seq data and a list of differentially expressed genes from wt and Sox2-deleted NSCs were downloaded from [8] (GEO accession number GSE90561). The biological functions of downregulated genes were investigated by gene ontology (GO) enrichment using the online tool WebGestalt (WEB-based Gene SeT AnaLysis Toolkit^3^) [15]; over-representation analysis (ORA) on biological processes was performed using as a reference set the list of genes overall expressed by either wt or Sox2-deleted NSCs. Only categories with a gene number between 100 and 2000 were considered, and the multiple test adjustment method was set to Benjamini–Hochberg (BH); other parameters were set as default. For further analyses, genes belonging to GO terms inherent to neuronal differentiation, development, maturation, function, and maintenance were extrapolated.

### 2.9. CUT&RUN

Raw reads were taken from our previously published datasets (ArrayExpress accession E-MTAB-9897) and trimmed using bbDUK (Bbtools-BBMap-Bushnell B.-sourceforge.net/projects/bbmap/). Trimmed reads were mapped to the mm10 genome using bowtie [16], with the settings -I 80 -X 400 -m 1 -v 3. Samtools [17] was used to create bamfiles, including a set of files mapping at or below 120 basepairs (“subnucleosomal reads”). Bedgraphs were created using bedtools [18]. Bedtools was also used to remove blacklisted regions. Peaks were called using SEACR [19] in relaxed mode, normalizing to negative control tracks. To create a stringent set of peaks, peaks from individual subnucleosomal tracks were intersected with consensus peaks from either AP1 and SOX2 and collected into a single track. Overlapping peaks were extracted using bedtools. Motifs underlying all peaks, and subnucleosomal peaks, were found using HOMER [20] using the masked mm10 genome and options -size given and -cpg. Peaks were annotated using GREAT [21], using default settings (basal plus extension mode). Lists of annotated peaks were intersected with RNA-seq data from [8]. Genes downregulated upon knockout of SOX2 were extracted using log2-fold change -1. STRING-db [22] was used to create interaction graphs of genes. Only edges found in experiments and databases at a confidence level of 0.5 were kept. The proteins annotated with GO term neuron differentiation in the largest consecutive cluster were extracted and re-graphed, allowing coexpression.

## 3. Results

### 3.1. Lentiviral Expression of Sox2 in Sox2-Deleted NSC Rescues Their Ability to Generate Neurons upon Induction of Differentiation

*Sox2*-deleted (Sox2-del) NSC, obtained from neonatal mice by Sox2 conditional deletion in vivo via Nestin-Cre [3], progressively loses the ability to self-renew as undifferentiated cells in in vitro culture, within about 5–9 passages [3]. We performed RNA-seq experiments, comparing three wild-types (WT) and three Sox2-deleted (Sox2-del) NSC cultures derived from independent mice; we performed the experiment at early passages, when growth kinetics of WT and Sox2-del were still similar. This analysis revealed a significant decrease in the expression of about 1000 genes in Sox2-del cells [8]. In *Sox2*-del NSC, *Socs3* and *Fos* mRNAs are strongly and highly significantly downregulated in the undifferentiated state (differentiation day 0, D0), as compared to their high expression in WT cells (Table 1) [8,9]. As *Socs3* and *Fos* mRNA re-expression in Sox2-deleted cells via viral transduction rescues long-term self-renewal [8,9], it is possible to obtain viable NSC populations lacking *Sox2*; this gives us the opportunity to examine the role of FOS and SOCS3 in cell differentiation.

Upon induction of in vitro NSC differentiation of the same cell cultures analyzed at D0, both *Socs3* and *Fos* mRNAs are strongly downregulated, in both WT and Sox2-del cells, at differentiation day 4 (D4) as measured by RNA-seq (Table 1); subsequently, upon addition of serum and mitogen removal at D4, the levels of *Socs3* and *Fos* are again moderately increased at D11. At D4 and D11, Fos mRNA levels were not significantly different between WT and Sox2-del NSC (FDR: 0.14 at D4, >0.2 at D11); *Socs3* mRNA levels did not significantly differ between WT and Sox2-del NSC at D4 (FDR: 0.09), but were slightly, and significantly, upregulated in Sox2-del NSC at D11 (FDR: 0.0006). In parallel, *Sox2* expression in WT NSC progressively slightly decreased between D0 and D11, by about 30% (Table 1).

**Figure 1:**
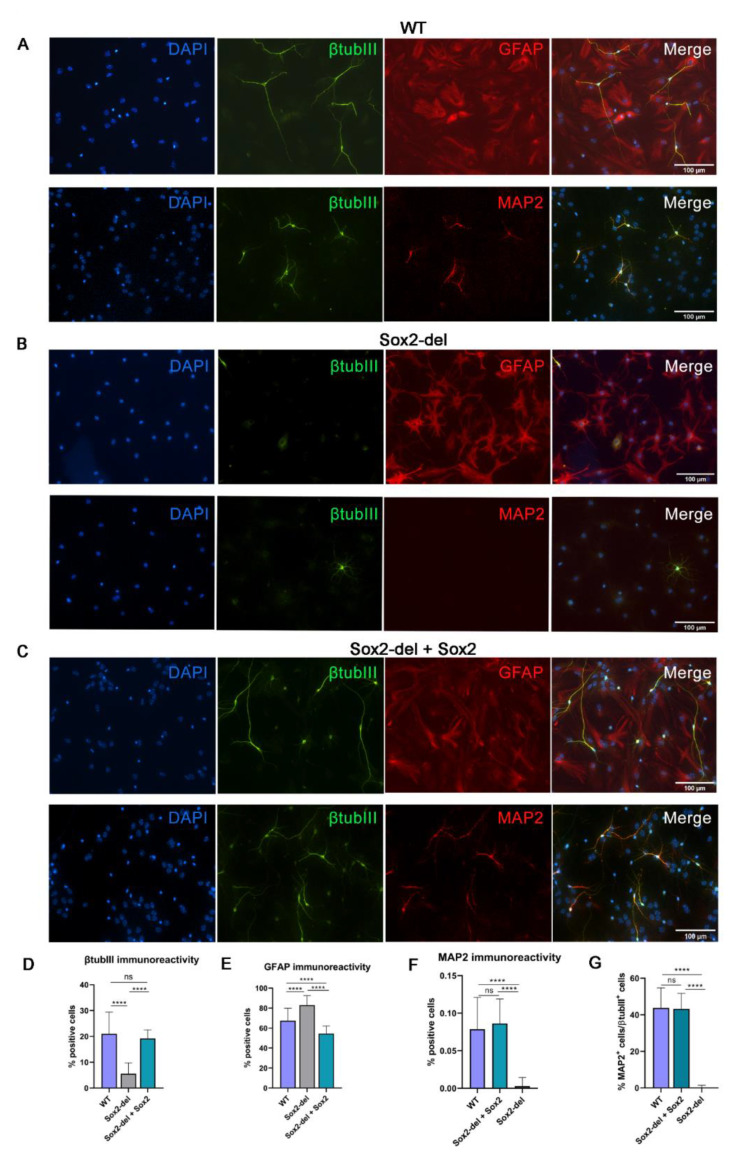
*Sox2* deletion impairs neuronal differentiation of NSC, and this is rescued by lentiviral *Sox2* re-expression. (**A**) Immunofluorescence (IF) for β-tubulinIII, a neuronal marker, GFAP, a glial marker, and MAP2, a marker of mature neurons in WT cells at D11. Top: β-tubulinIII positive cells show long arborizations and mature neuronal morphology. Bottom: all β-tubulinIII positive cells are also MAP2 positive. (**B**) IF for β-tubulinIII, GFAP and MAP2 IF in Sox2-del cells at D11. IF shows few β-tubulinIII positive cells with an immature morphology; some cells also show β-tubulinIII/GFAP double positivity. MAP2-positive cells are absent. (**C**) β-tubulinIII, GFAP, and MAP2 IF in *Sox2*-transduced Sox2-del cells (Sox2-del + Sox2) at D11. (**D**–**F**) Histograms show the percentage of cells positive for β-tubulinIII, GFAP and MAP2, respectively, compared to the total cell number. (**G**) Histograms show the percentage of MAP2-positive cells/β-tubIII-positive cells. (**D**,**E**) Results are the mean of counting 16–20 fields in each of *n* = 7 independent differentiation experiments conducted on 3 different WT samples (from 3 WT mice) and 3 different Sox2-del samples (from 3 Sox2-del mice); (**F**,**G**) Results are the mean of 16–19 fields counted in *n* = 2 independent differentiation experiments, each conducted on two WT and two Sox2-del samples. Statistical significance was assessed by Brown Forsythe ANOVA and Dunnet T3 test (**** *p* < 0.0001; ns: not significant).

We first tested in vitro differentiation to neurons and glia of WT and Sox2-del cells (Figure 1 and Figure S1). Morphological and immunofluorescence analysis demonstrated that, in differentiated Sox2-del cells, the proportion of neuronal cells (as identified by extended neurites and beta-tubulinIII positivity) was strongly decreased (Figure 1A,B,D). MAP2 positivity, marking more mature neuronal cells, was absent in Sox2-del cells, although it was present in control WT cells (Figure 1A,B,F). In contrast to the neuronal population, glial cells, marked by GFAP expression, displayed a normal morphology in Sox2-del cells. The proportion of glial cells was increased in Sox-del cells, mirroring the decrease in the proportion of neuronal cells (Figure 1A,B,E). These results are reminiscent of those obtained by Cavallaro et al. [10], using NSC from a *Sox2* hypomorphic mutant, expressing approximately 30% of the normal (wild type) *Sox2* level.

We then re-expressed *Sox2* in Sox2-del NSC via a lentiviral vector (Figure S2), rescuing long-term growth and generating a population that expresses *Sox2* in almost all NSCs [9]. When these *Sox2*-transduced, Sox2-del NSC were differentiated as described above, the number of neurons obtained at D11 returned to essentially normal levels, when compared to WT cells (Figure 1A–D). In addition, their morphology appeared to recover “maturity”, as indicated by arborization and MAP2 positivity (Figure 1A–C,F,G); the number of “mature” cells in the *Sox2*-transduced, Sox2-del cells fully recovered the numbers seen in WT cells (Figure 1D,F,G). The proportion of glial cells in *Sox2*-del *Sox2*-transduced cells was significantly reduced in comparison to *Sox2*-del cells as expected, due to the increased proportion of neuronal cells caused by *Sox2* transduction, and possibly also to some defect in gliogenesis (Figure 1A–C,E); indeed, *SOX2* acts as a *GFAP* repressor in cotransfection experiments [10]. These results confirm and extend the ability of SOX2 to rescue the defective neuronal maturation observed in *Sox2*-deficient hypomorphic NSC [10].

### 3.2. Lentiviral Expression of Fos in Sox2-del NSC Rescues Their Ability to Generate Neurons upon Induction of Differentiation

**Figure 2:**
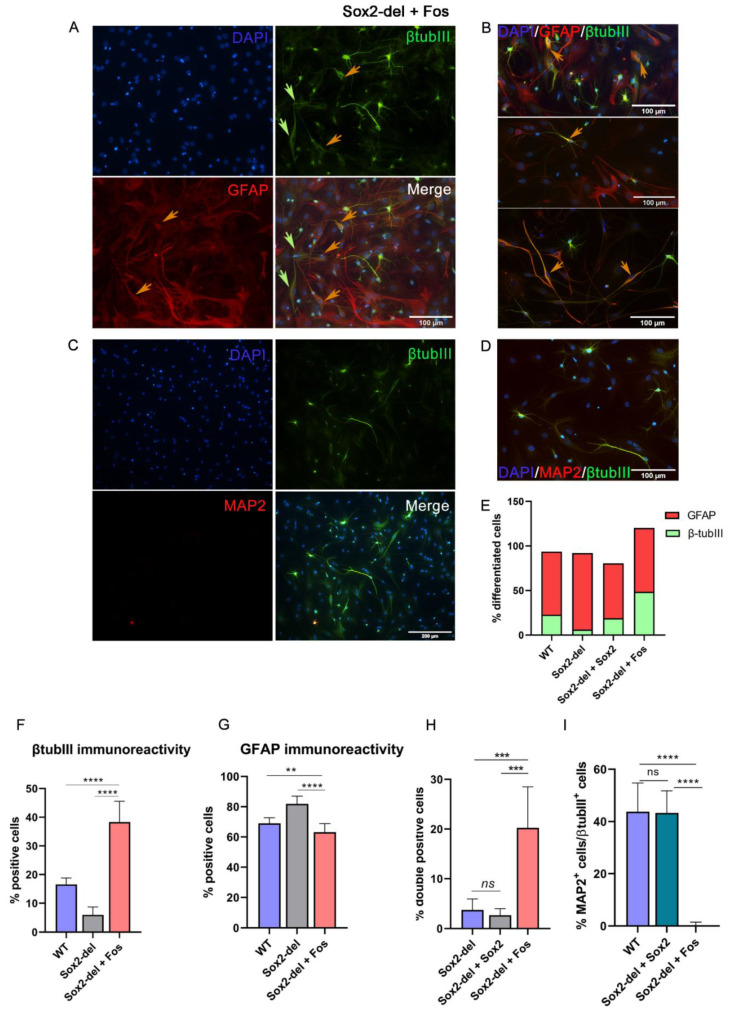
Lentiviral expression of *Fos* in *Sox2*-deleted NSC rescues their ability to generate neurons upon induction of differentiation. IF for β-tubulinIII, GFAP, and MAP2 in *Fos*-transduced Sox2-del cells (Sox2-del + Fos) at D11. (**A**) Fos overexpression rescues the differentiation into neurons (β-tubulinIII-positive cells); some cells are positive for both β-tubulinIII and GFAP (orange arrows), and some β-tubulinIII-positive cells have a somewhat “glial” morphology (green arrows). (**B**) Double-positive cells have different morphologies: top panel, glial-like cells; middle panel, neuronal-like cells; bottom panel, poorly defined morphology. (**C**,**D**) Absence of MAP2 staining in Sox2-del, Fos-transduced differentiated cells. (**E**) Histograms show the percentage of differentiated cells out of the total; in the Sox2-del + Fos population the total % of β-tubIII + GFAP-positive cells is greater than 100%, indicating the existence of double-positive cells. (**F**,**G**) Percentage of β-tubIII, GFAP, and MAP2-positive cells/total cell number. (**H**) Percentage of double-positive cells/total cell number in Sox2-del, Sox2-del + Sox2, and Sox2-del + Fos samples. Fos overexpressing cells often show double positivity for β-tubIII and GFAP. (**I**) Percentage of MAP2-positive cells/β-tubIII-positive cells. Results are the mean of 16–20 fields counted in *n* = 2 independent differentiation experiments. Statistical significance was assessed by Brown Forsythe ANOVA and Dunnet T3 test (**** *p* < 0.0001; *** *p* < 0.001; ** *p* < 0.01; ns: not significant).

*Fos* expression is strongly reduced in Sox2-del NSC, and *Fos* is a direct target of SOX2 itself [8,9]. Of note, we have previously shown that *Fos* expression by lentiviral transduction rescues long-term self-renewal of undifferentiated Sox2-del NSC [9]. We obtained populations of *Fos*-transduced Sox2-del cells (Figure S2), which, after 6–7 passages, mostly consisted of lentivirally transduced cells, presumably due to positive selection for *Fos* expression. As *Fos* is downstream to SOX2, we asked whether *Fos* might be a mediator of the SOX2-induced rescue of neuronal generation shown in Figure 1, by studying the differentiation of *Fos*-transduced *Sox2*-deleted NSC. Figure 2 shows that, indeed, *Fos* induces a strong increase in the number of cells expressing the beta-tubulin III neuronal marker, even exceeding (up to 40–50%) the proportion of beta-tubulin-III-positive cells observed in WT cells (Figure 2A–C,E,F); conversely, the fraction of GFAP-positive cells was moderately decreased (Figure 2G). Importantly, when stained both with anti-GFAP and with anti-beta-tubulin-III antibodies, many cells showed a double positivity (Figure 2A,B), so that the number of beta-tubulin-III-positive plus GFAP-positive cells was well above the total number of cells (Figure 2E). Interestingly, MAP2 staining was not observed in Sox2-del, *Fos*-transduced differentiated cells (Figure 2C,I). Looking at cell morphology, most cells positive for beta-tubulin III showed a neuronal appearance, similar to that seen with WT cells, whereas the majority of doubly stained (beta-tubulin + GFAP) cells had a poorly defined morphology, neither clearly neuronal nor glial (Figure 2B). Overall, these results indicate that *Fos* overexpression was able to stimulate the initial neuronal differentiation of bipotent neuronal/glial progenitors, but fails to induce a more advanced neuronal differentiation program, with the appearance of mature neuronal markers (MAP2) and complete extinction of GFAP expression.

### 3.3. Lentiviral Expression of Socs3 in Sox2-del NSC Inhibits the Genesis of Glial Cells upon Induction of Differentiation

**Figure 3:**
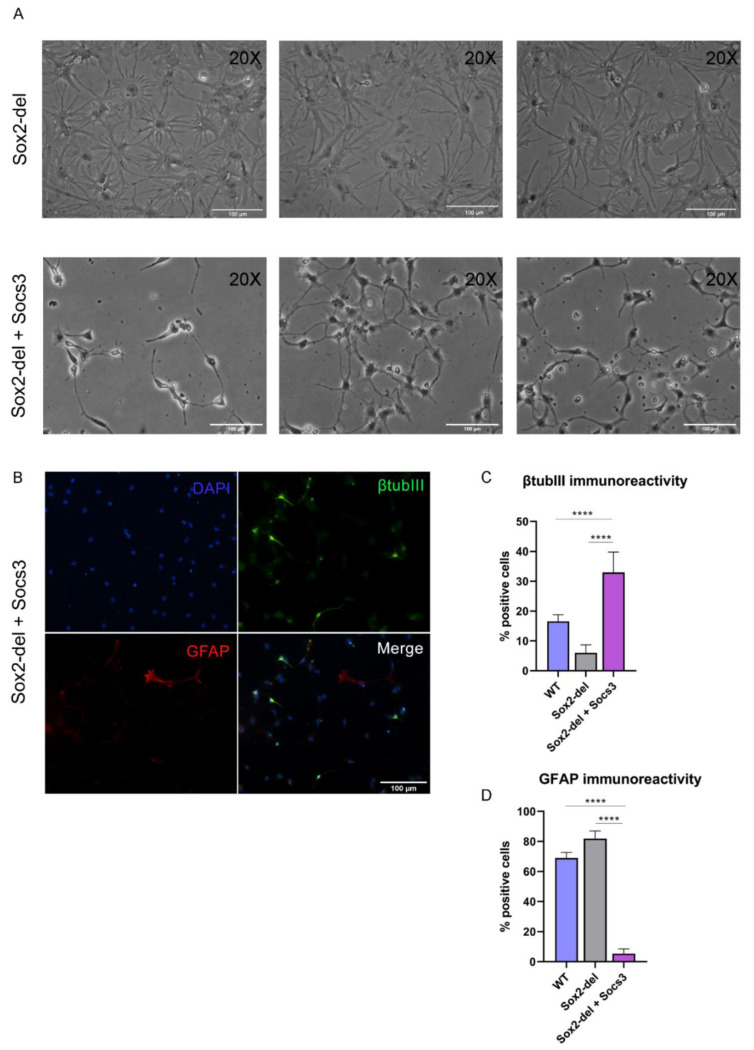
Lentiviral expression of *Socs3* in Sox2-del NSC inhibits gliogenesis upon induction of differentiation. (**A**) Bright-field microscopy of Sox2-del cells (top row) and *Socs3*-transduced Sox2-del cells (Sox2-del + Socs3; bottom row) at differentiation day 11. Sox2-del + Socs3 cells (bottom) are strongly abnormal, and no longer display glial morphology (**B**). IF of Sox2-del + Socs3 cells; most β-tubulinIII-positive cells, although present, do not show neuronal morphology and most of them are weakly stained. GFAP-positivity is essentially absent (compare to Figure 1). (**C**,**D**) Histograms show the percentage of β-tubulinIII and GFAP-positive cells/total cell number. Results are the mean of 16 fields counted in *n* = 2 independent differentiation experiments. Statistical significance was assessed by Brown Forsythe ANOVA and Dunnet T3 test (**** *p* < 0.0001; ns: not significant).

*Socs3* expression is extremely reduced in Sox2-del NSC; re-expression of *Socs3* by lentiviral transduction (Figure S2) allows Sox2-del NSC to maintain long-term self-renewal [8]. We induced differentiation of long-term proliferating *Socs3*-transduced, Sox2-del NSC: this resulted in a dramatic decrease of glial cells, as indicated by morphological observations and GFAP staining (Figure 3A,B). As in Sox2-del cells (Figure 1), the number of well-differentiated neurons was strongly decreased (Figure 3B,C); note, however, that a large number of weakly beta-tubulin-III-positive cells, not resembling neurons, were detected in *Socs3*-transduced Sox2-del cells. We conclude that *Socs3* overexpression antagonizes glial differentiation, but is unable to rescue neuronal differentiation; the high number of beta-tubulin-III-positive cells observed in Sox2-del cells transduced with *Socs3* might represent bipotent glial/neuronal progenitors that are arrested at a very early stage of differentiation, before resolving their fate into either neuron or glia (due to the combined effect of lack of *Sox2* and inhibition of gliogenesis by *Socs3*).

### 3.4. RNA-seq Demonstrates That Genes Downregulated in Undifferentiated Sox2-del NSC Are Enriched in Genes Involved in Neuronal Differentiation

In Sox2-del NSC, approximately 1000 genes are downregulated in the undifferentiated state [8]. We analyzed, by over-representation analysis (ORA), the gene ontology (GO) terms more significantly enriched among the genes downregulated in Sox2-del cells, as compared to the total population of genes expressed in WT NSC. Figure S3 shows that the most significantly enriched GO categories among genes downregulated in Sox2-del cells include genes whose function is related to various aspects of neuronal differentiation; a list of these genes is provided in Table S2. This result is somewhat unexpected, and suggests the intriguing notion that SOX2 might prime, already in undifferentiated cells, the initial expression of genes related to the neuronal fate.

### 3.5. CUT&RUN Reveals Broad Genome-Wide Co-Occupancy of SOX2 and the AP1 Complex in NSC

**Figure 4:**
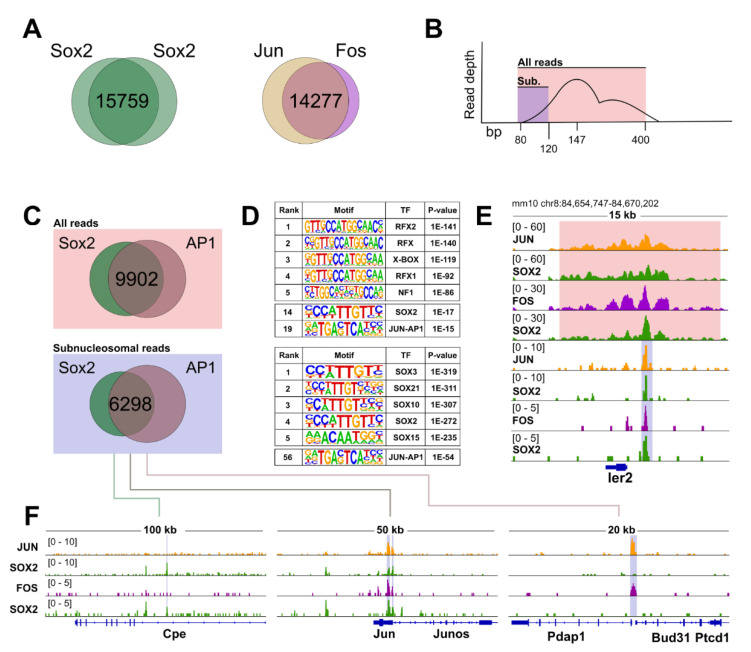
Genome-wide binding of SOX2 and AP1. CUT&RUN of AP-1 and SOX2. (**A**) Peaks were called for the SOX2, FOS, and JUN datasets, revealing overlaps of 15,759, and 14,277 peaks, respectively. (**B**) Schematic representation of our in-silico size selection approach. The semi-transparent boxes indicate the ranges of read size (expressed in base pairs, bp) considered. (**C**) Overlapping peaks called from all reads with a cut-off of 400 base pairs max insert-size yielded 9902 shared peaks. Enriching for subnucleosomal reads/peaks reduced this number to 6298. (**D**) The top five consensus sequences identified from the motif analysis using HOMER, ranked based on increasing p-value (1E represents standard notation for powers of 10; e.g., 1E-141 = 1 × 10^−141^). Representative motifs for SOX proteins and AP1 are added below in the same table. Their ranking is indicated with a number on the left. (**E**) IGV tracks of the four samples, showing the different enrichment profiles of the supra- and sub-nucleosomally enriched peaks. (**F**) Representative SOX2 only (left), SOX2/AP1 shared (middle), and AP1 only (right) peaks.

Our previous results are suggestive of the possibility that SOX2, together with FOS (and possibly JUN, forming with FOS the AP1 complex) acts to regulate genes important for NSC differentiation. To test this, we analyzed in detail the CUT&RUN genome-wide chromatin binding analysis of SOX2, FOS, and JUN previously performed in NSC [9]. We derived two independent lists of high-confidence binding regions (15.759 for SOX2 and 14.277 for AP1, Figure 4), by considering the peaks obtained with the corresponding antibodies (Figure 4A). Strikingly, we found that when the SOX2 and the AP1 high-confidence peaks were compared, many of the resulting genomic locations appeared to be shared, indicating possible co-occupancy of several loci, genome-wide, by SOX2 and AP1 (Figure 4C). SOX2 mediates functional long-range interactions between regulatory regions required for gene expression [8]. We reasoned that CUT&RUN might detect regulatory regions that come in physical proximity via looping with the SOX2-bound loci and that not all the peaks identified are therefore caused by direct SOX2 or AP1 binding to the DNA. Therefore, we aimed at identifying primary SOX2 and AP1 binding sites by in silico size-selection of the sub-nucleosomal fraction of the obtained reads (Figure 4B,E; Table S3); this approach was previously shown to enrich for those regions that most likely represent specific transcription factors footprints [23]. This stringent analytical pipeline yielded a group of 6298 loci that are more likely to be directly bound by both SOX2 and AP1 (Figure 4C,F; Table S3). Consistently, motif analysis performed on this set of genomic regions displayed prominent enrichment for SOX proteins, and also increased the statistical confidence in the identification of the bZIP (leucine zipper, such as FOS and JUN) consensus sequence (Figure 4D, bottom panel). Taken together, these analyses unearth a previously neglected, broad genomic interplay between SOX2 and the AP1 complex in NSC.

### 3.6. The SOX2/AP1 Duet Regulates a Large Fraction of Genes Expressed in NSC

**Figure 5:**
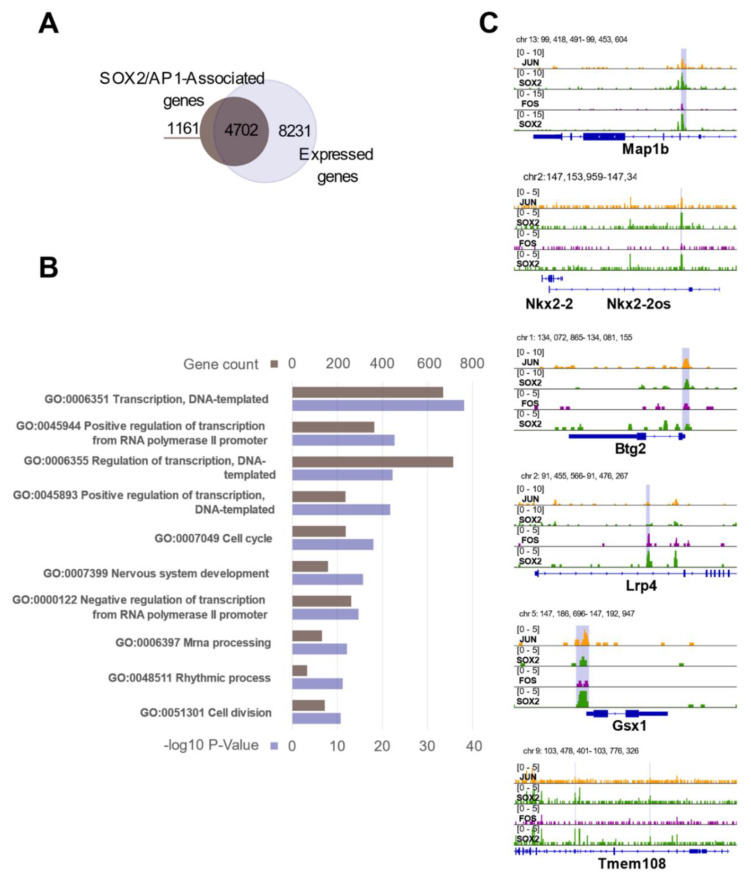
SOX2 and AP1 co-regulate a plethora of biological processes. (**A**) Subnucleosomally enriched co-bound peaks were annotated using GREAT, and overlayed with all genes expressed in NSC (mean TPM > 1) [8]. (**B**) Functional enrichments (biological process, DAVID) were performed on the overlapping genes, and the top 10 hits (rising p-value) are shown. See also Tables S3 and S4. (**C**) AP1 and SOX2 co-occupy many loci.

Above, we have derived a set of 6298 genomic regions that are likely bound by both SOX2 and the AP1 complex in NSC. We next asked whether the observed SOX2/AP1 co-occupancy corresponds to the transcriptional regulation of specific sets of genes in NSC. We annotated the 6298 common SOX2/AP1 peaks, relating them to specific genes using GREAT [21], with each gene assigned a basal regulatory domain of a minimum distance upstream (5 kilobases) and downstream (1 kilobase) of the transcription start site (TSS). We obtained a list of 5863 genes that are likely regulated by the observed SOX2 and AP1 association (Figure 5A, SOX2/AP1-associated genes). In parallel, we derived a list of 12,933 genes that are expressed in NSC, as they displayed a TPM value above 1 in our RNA-seq experiments [8] (Figure 5A, Expressed genes). Of note, a large fraction of 4702 genes, which corresponds to ca. 36% of all the expressed genes in undifferentiated NSC, are associated with SOX2 and AP1 occupancy (Figure 5A). Gene ontology (GO) analysis of this group of genes yielded broad categories which, when ranked from lowest false discovery rates (FDR), were enriched in terms such as regulation of cellular, developmental, and metabolic processes (Figure 5B; Table S4). These observations thrust forward the important notion that SOX2 and AP1 act together as a functional duet, likely necessary for general aspects of gene regulation in NSC. Focusing on genes and immediately surrounding regions (Figure 5C), we note that the observed binding of both SOX2 and AP1 was either on gene promoters (e.g., *Btg2*, *Gsx1*), or within introns (e.g., *Lrp4*), or within sequences adjacent to the gene (e.g., *Nkx2-2*), within the flanking *Nkx2-2os* region (Figure 5C). Interestingly, the non-promoter peaks in *Map1b, Lrp4, Tmem108,* and in the region flanking *Nkx2-2* all carry epigenetic enhancer marks [8]; further, the chromatin region where the *Lrp4* intronic peak and the peak adjacent to *Nkx2-2* are located is connected, in ChIA-PET experiments, to the respective gene promoter by long-range interactions [8]. These results point to a role of FOS (as well as SOX2) at both promoter and enhancer regions.

### 3.7. SOX2 and the AP1 Complex Regulate Genes Involved in Neuronal Differentiation

**Figure 6:**
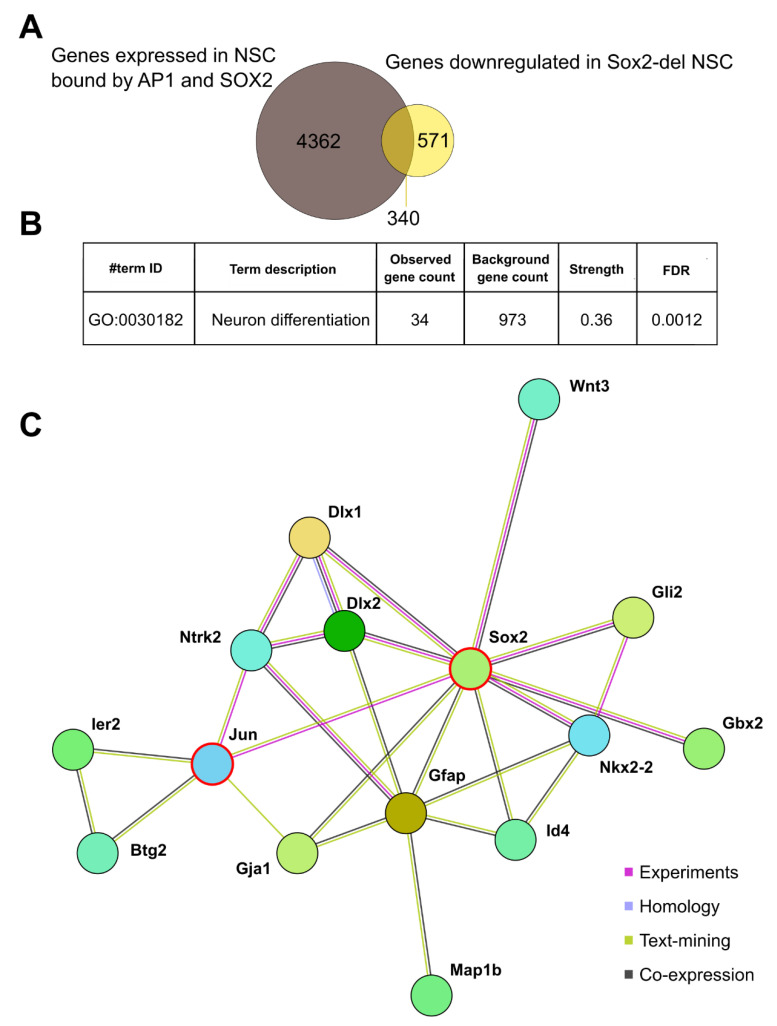
SOX2 and AP1 co-regulate genes involved in neuronal differentiation. (**A**) Expressed genes bound by AP1 and Sox2 were overlayed with genes that are significantly downregulated (fold change > 2; TPM > 1) upon knockout of *Sox2* in NSC [8] (see also Table S5). (**B**) An interaction network was generated using STRING, and a gene ontology enrichment was performed, showing 34 genes with the GO:term Neuron differentiation. The largest cluster was isolated, and in that cluster were 18 genes with the GO term neuronal differentiation (see also Table S6). (**C**) Another gene-interaction network was generated, which represents a putative regulatory core of players governed by AP-1 and SOX2.

A prominent feature of the Sox2-del NSC is their strongly defective production of neurons upon differentiation (Figure 1 and Figure 2). This neuronal “incompetence” is largely rescued by either *Sox2* or *Fos* overexpression, hinting at the intriguing possibility that the concerted action of SOX2 and AP1 is relevant for the expression of genes involved in neuronal differentiation. To test this, we considered the genes that were differentially regulated in NSC upon *Sox2* deletion [8] and their overlap with genes annotated to peaks using GREAT annotation, and extrapolated a short-list of 340 downregulated genes whose expression is, by definition, dependent on SOX2 presence (Figure 6A; Table S5). Notably, this group contains genes that have been previously described as relevant for neuronal differentiation. We compared this list of SOX2 targets with our list of SOX2 and AP1 co-bound genes, to determine if they were regulated by SOX2/AP1 interplay, and we found that a consistent number of them displayed concomitant binding events of SOX2 and AP1 in their genomic loci. Among these, notable examples are *Btg2* [24,25,26], *Lrp4* [27], *Map1b* [28,29,30], *Tmem* 108 [31,32], *Nkx2-2* [33,34,35], *Wnt3* [36], *Gli2* [37], *Egr2/Egr* [38], *Plxnb3* [39], *Gbx2* [40], *Gsx1* [41,42], *Jun* [43], *Tenm4* [44] (some of them are shown as example in Figure 5B). Strikingly, when these 340 downregulated genes were analyzed using the STRING database, which matches functional interactions across protein networks by unbiased literature mining [45], we uncovered a SOX2/AP1-centered regulatory network composed of neuronal players (Figure 6B,C and Figure S4; Table S6). This indicates that SOX2 might be actively involved in the regulation of differentiation events by interplay with the AP1 complex.

## 4. Discussion

### 4.1. Fos Overexpression in Sox2-del Cells Favours Initial Neuronal Differentiation of a Glial/Neuronal Progenitor

**Figure 7:**
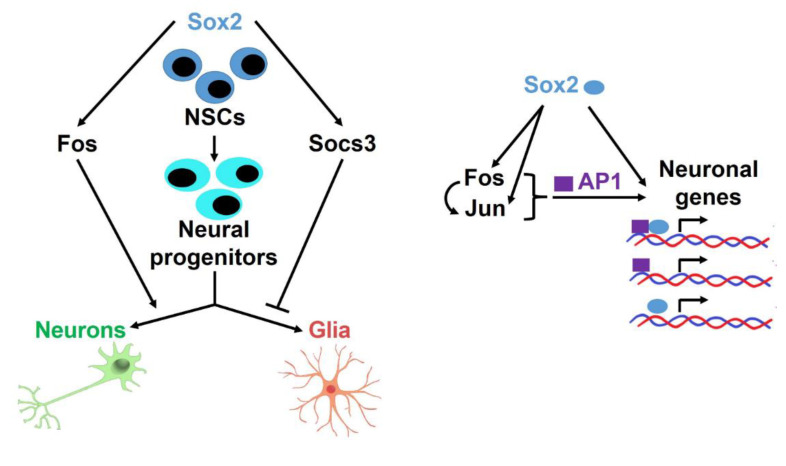
A simplified model for the roles of *Sox2*, *Fos*, and *Socs3* in NSC differentiation.

Recently, we showed that *Fos* overexpression in Sox2-del NSC fully rescued their defect in long-term proliferation [9]. As *Fos* is very highly expressed in NSC, and it is strongly downregulated in Sox2-del NSC, we proposed a model whereby SOX2 maintains NSC self-renewal, at least in part, via activation of downstream effectors, such as FOS, JUN, and SOCS3. A potential implication of this model is that FOS might share with SOX2 a proportion of target genes. Another study [10] has previously demonstrated that hypomorphic *Sox2* mutant NSC, expressing only 30% of the wild type *Sox2* levels, had significant defects in the in vitro formation of neurons upon differentiation. In the present study, we confirmed that the absence of *Sox2* (complete deletion) strongly impacted neuronal production and showed that *Sox2* re-expression rescued the neuronal phenotype (Figure 1 and Figure 2). We, therefore, tested the hypothesis that also *Fos* overexpression in Sox2-del NSC might rescue the defect in neuronal differentiation. As shown in Figure 2, long-term proliferating Sox2-del NSC, obtained following *Fos* overexpression [9], showed recovery of the ability to initiate neuronal differentiation, generating normal numbers of neurons of differentiated morphology and expressing beta-tubulin III, although not the more mature marker MAP2. Interestingly, a large number of cells additionally show mixed glial/neuronal morphology and coexpression of GFAP and beta-tubulin III, indicating that FOS might bias the differentiation of bipotent glial-neuronal progenitors towards the neuronal lineage (Figure 2). These bipotent progenitors are rarely, if at all, observed in wild-type differentiated cells, but are present in differentiated *Sox2*-deficient cells ([10]; present paper, Figure 2). Thus, we propose that, in the absence of SOX2, NSC differentiation is often arrested at a bipotent glial/neuronal progenitor level; by overexpressing *Fos*, cells may be diverted from the glial fate towards the neuronal fate, and a proportion of them may initiate the correct neuronal differentiation (see model in Figure 7).

### 4.2. FOS Binds, in NSC, to a Large Proportion of Expressed Genes Close to SOX2-Bound Regions

We used CUT&RUN to assay for FOS, JUN (forming the AP1 complex) and SOX2 binding to the chromatin of wild-type NSC. The results clearly showed that FOS, in addition to binding to some specific targets, often binds to common targets also recognized by SOX2 (36% of all expressed genes, Figure 5). Importantly, a high frequency of FOS binding together with SOX2 is observed in a fraction of genes that are downregulated in Sox2-del NSC and are bound by SOX2 in wild-type NSC (Figure 6). This observation supports the idea that FOS might contribute to the rescue of neuronal differentiation by activating (at least to some extent) genes that are downregulated in the absence of *Sox2*. Indeed, it is interesting that several genes that are known to be essential for correct differentiation of various neuronal types are among the common FOS/SOX2 targets (Figure 4 and Figure 5 and related references in text); some of these, when mutated, cause brain disease in humans. We would like to hypothesize that neuronal genes already expressed in NSC are “primed” by SOX2 and FOS in view of a full activation at more advanced stages of neuronal differentiation.

The level of FOS expression in NSC is exceedingly high, suggesting broad functions in these cells. Recent models [46,47] propose that specific “anchor” DNA sequences initially recruit defined transcription factors. These factors increase the accessibility of specific chromatin regions, acting as a core around which other factors aggregate in a way that is independent of DNA sequence. This could occur via protein–protein interactions and chromatin loops, which might connect several distant regulatory elements. In addition, it was shown that FOS/JUN selects enhancers together with cell-type-specific transcription factors by binding to enhancers in the context of nucleosomes, and by recruiting the SWI/SNF chromatin-remodeling complex to establish an accessible chromatin region [48]. Interestingly, our efforts aimed at identifying the genomic regions that most likely represent the direct SOX2/AP1 targets displayed prominent enrichment of SOX binding motifs (Figure 4B). It is tantalizing to hypothesize that, in NSC, SOX2 is responsible for the recruitment of AP1 on most of the common regulatory regions. SOX2 is, in fact, essential for maintaining promoter/enhancer loops, and this might recruit distant AP1-bound regulatory regions in the proximity of promoters [8]. Accordingly, several of the SOX2/AP1 peaks are located in gene-distant or intronic regions (Figure 4 and Figure 5), raising the possibility that these are neural- or neuronal-specific AP1-dependent enhancers that interact with gene promoters via SOX2-mediated DNA-looping [8]. This seems to be the case with some genes shown as examples in Figure 5C, *Lrp4*, and *Nkx2-2.*

### 4.3. SOX Transcription Factors Orchestrate Signalling Pathways by Promiscuous Interactions

The family of SOX transcription factors has been repeatedly implicated in the regulation of signaling cascades relevant for the specification of neural structures. For example, ectopic activation of *Sox2* can specify sensory cell progenitors of the mouse cochlea, similarly to Notch pathway activation, possibly suggesting that neuronal programs might be activated by redundant mechanisms [49]. Moreover, SOX2 was found to regulate neurogenesis from human neural progenitor cells by inhibiting canonical Wnt signaling [50]. In this context, SOX2 might antagonize the Wnt pathway to maintain a neurogenic fate [51]. On the other hand, a recent report uncovered a dual role for SOX2, in which high SOX2 displaces nucleosomes to recruit the Wnt effectors TCF/LEF and β-catenin and to maintain the expression of pluripotency genes, while loss of SOX2 would allow WNT-induced mesodermal differentiation (Blassberg et al., BioRxiv; doi:10.1101/2020.12.29.424684). Notably, it is precisely during mesoderm formation that one of the best-documented cases of the SOX-WNT interactions occurs, exemplified by the SOX17 genome-wide co-occupancy with β-catenin [52]. This indicates that the SOX factors might govern the tissue-specific action of Wnt signaling not only in embryonic stem cells or neural progenitors, but more in general during development [53].

Importantly, our work reveals that a previously overlooked joint action between SOX2 and the AP1 complex, which is an important effector of signaling cascades such as the MAPK/ERK/RAS pathway [54], might be a novel constituent for the neuronal differentiation program. It remains, however, to be elucidated how *Fos* overexpression can rescue the loss of *Sox2*. We speculate that, in the absence of SOX2, other SOX proteins might “prime” the transcriptional activity of AP1 by tethering FOS (and/or JUN) on the relevant regulatory regions. Notably, recent reports showed that the sub-family of SOXB1 transcription factors, of which SOX2 is a member together with SOX1 and SOX3, bind to overlapping sites in neural cells chromatin [55] and act redundantly to maintain naïve pluripotency in embryonic stem cells [56]. This is additionally supported by the presence of motifs of other SOX proteins in our high-confidence SOX2 CUT&RUN peaks (Figure 4B, bottom panel) and might provide a mechanistic explanation of how FOS rescues SOX2 deficiency. Whether other SOXB1 proteins engage in a genome-wide functional interplay together with AP1 remains to be tested.

### 4.4. A Network of Interactions Directing Neuronal versus Glial Differentiation

In this work, we describe the opposite roles of FOS, a transcription factor, and of SOCS3, a modulator of cytokine-dependent signaling, on the differentiation of neural progenitors into neurons and glia, respectively. Both *Fos* and *Socs3* are regulated by SOX2, and their levels are strongly decreased in *Sox2*-del cells; both *Fos* and *Socs3*, when overexpressed, rescue long-term proliferation of *Sox2*-del cells. In addition, FOS activates *Socs3* in different cell types, including pro-B lymphocytes, breast cancer cells, and NSC [9,57,58,59,60]. The experiments described here show that FOS, in addition to complementing SOX2 absence in maintaining long-term self-renewal [9], also contributes to improving neuronal differentiation. *Fos* overexpression increases (initial) neuronal differentiation at the expense of glial differentiation (Figure 2). Interestingly, it was reported that the knockout of *Fos* causes a neuronal differentiation defect during mouse brain development [61,62]. On the other hand, *Socs3* strongly inhibits glial differentiation (Figure 3). SOCS3 is a negative regulator of cytokine signaling based on the JAK-STAT pathway; STAT3 eventually activates SOCS3, which in turn negatively regulates STAT3, attenuating the effect of JAK-STAT activation [60]. Factors such as leukemia inhibitory factor (LIF), BMP2, and others have been implicated in gliogenesis dependent on the SOCS3-attenuated JAK-STAT pathway [60,63]. Accordingly, the knockout of SOCS3 causes inappropriately increased gliogenesis in vitro and in vivo [60,63]; conversely, SOCS3 overexpression inhibits astrogliogenesis [59], and LIF treatment efficiently promotes astrogliogenesis in the absence (knockout) of Socs3 [60,63]. In NSC lacking *Sox2* and overexpressing *Socs3*, both glial and neuronal differentiation are strongly impaired, due on one side to excess *Socs3* expression, which represses gliogenesis, on the other side to the lack of *Sox2* that prevents the differentiation into neurons of the glial/neuronal bipotent progenitors (Figure 3 and Figure 7). Thus, SOX2 activates two genes, *Fos* and *Socs3*, whose balanced regulation is necessary to define the appropriate differentiation of neurons versus glia.

Further, SOX2 regulates the expression of JUN, together with that of FOS, which in turn also binds to the Jun gene (Figure 4), presumably regulating it and creating a virtuous circle (Figure 7).

Finally, the human *Sox2* gene homolog *SOX2*, together with *FOXP2* (whose mutation causes language impairment), *GLI3* (part of the network in Figure 6), RCAN1 (involved in the pathogenesis of Down’s syndrome) and other genes, belong to a set of genes whose enhancers or promoters harbor modern human single-nucleotide changes that appeared after the split from the Neanderthal/Denisovan lineage, and have been proposed to contribute to modern human-specific characteristics [64]. In mice, SOX2 directly binds to FOXP2, GLI3, and RCAN1 regulatory elements [8], raising the intriguing possibility that changes in SOX2/AP1 co-binding may contribute to the evolution of human-specific gene regulation.

## Figures and Tables

**Table 1:** Levels of *Fos* (A), *Socs3* (B), *and Sox2* (C) mRNA, in wild-type and Sox2-del cells during NSC differentiation (D0, D4, D11). Three wild-type (WT) and three *Sox2* mutant (Sox2-del) mice were used to generate NSC cultures that were then analyzed at D0, D4, and D11 by RNA-seq. TPM, transcripts per million. D0 values are from our previous work [8]; D4 and D11 genome-wide RNA-seq data are reported in Table S1. The slight increase of the ratio of Sox2 mRNA in Sox2-del versus WT at D4 and D11 might be due to preferential proliferation at early stages of differentiation, or better survival, of rare Sox2-positive cells present at P0.

	*Fos* (TPM Values)						
	WT				Sox2-del		
Mouse number	1		2	3	1	2	3
D0	3430.60		2984.97	2189.25	978.08	259.14	1218.42
D4	14.52		4.38	7.48	5.28	4.92	4.81
D11	22.80		58.71	32.46	29.79	64.64	35.61
	***Socs3* (TPM Values)**						
	**WT**				**Sox2-del**		
Mouse number	1	2		3	1	2	3
D0	235.80	336.16		191.33	27.50	11.18	49.28
D4	9.65	4.29		4.33	3.30	3.69	1.13
D11	20.20	19.08		21.81	41.68	50.67	44.26
	***Sox2* (TPM Values)**						
	**WT**				**Sox2-del**		
Mouse number	1		2	3	1	2	3
D0	495.40		306.24	247.93	2.59	6.64	3.45
D4	332.73		151.27	307.37	5.00	21.53	8.02
D11	200.03		224.55	242.31	11.84	26.34	17.22

## Data Availability

The CUT&RUN experiment has been deposited at ArrayExpress accession E-MTAB-9897. The RNA-seq raw data can be found in GEO under accession number GSE90561. The other data are available from the corresponding authors upon request.

## Supplementary Materials

The following are available online at https://www.mdpi.com/article/10.3390/cells10071757/s1, Figure S1: Secondary antibody-only control, Figure S2: RT-PCR quantitation of Sox2, Fos and Socs3 mRNA in WT, Sox2-del and Sox2-del NSC transduced with Sox2, or Fos or Socs3-expressing lentiviruses. qRT-PCR was performed in triplicate, Figure S3: GO-biological process analysis for genes downregulated in undifferentiated Sox2-del NSC, Figure S4: The whole interaction network from Figure 6, showing the biggest network using MCL 1.1, Table S1: RNA-Seq Expression Data for WT and Sox2-del NSC at D4 and D11, Table S2: Lists of genes belonging to each GO term in Figure S2 are given (sheet: enriched gene set GO analysis), Table S3. GREAT annotation of subnucleosomally enriched peaks shared between SOX2 and AP1 (.xcl file), Table S4. DAVID Gene Ontology analysis of genes bound by SOX2 and AP1 that are expressed in NSC (.xcl file), Table S5. Genes bound by SOX2 and AP1 and downregulated in SOX2-deleted NSC (.xcl file), Table S6. STRING Gene Ontologies for genes bound by SOX2 and AP1 and downregulated in SOX2-deleted NSC (.xcl file).
