# Supplementary material for: FOS Rescues Neuronal Differentiation of Sox2-Deleted Neural Stem Cells by Genome-Wide Regulation of Common SOX2 and AP1(FOS-JUN) Target Genes
Source: Cells. 2021 Jul 12;10(7):1757. doi: 10.3390/cells10071757 (PMC8303191; doi:10.3390/cells10071757)
Supplement: Supplementary file 1 [file cells-10-01757-s001.zip › cells-1264544 suppl_R1.pdf]

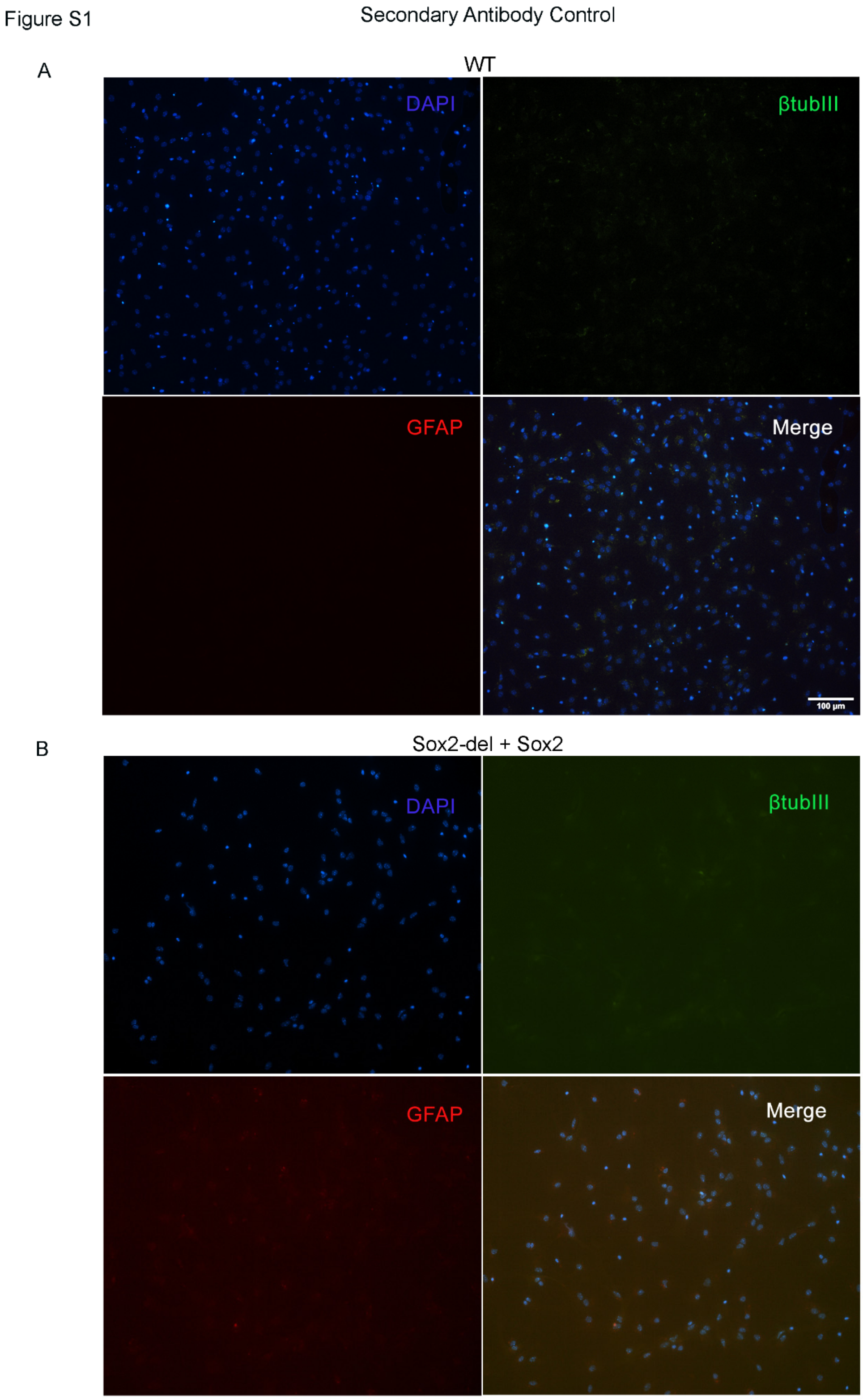

Figure S1. Secondary antibody-only control. A-B) Samples were incubated with antibody dilution buffer without the primary antibody, followed by incubation with secondary antibodies normally used to detect the anti- β-tubulinIII (green) or anti-GFAP (red) primary antibodies; no signal is detected, confirming that the specificity of the IF in Figs. 1-3.

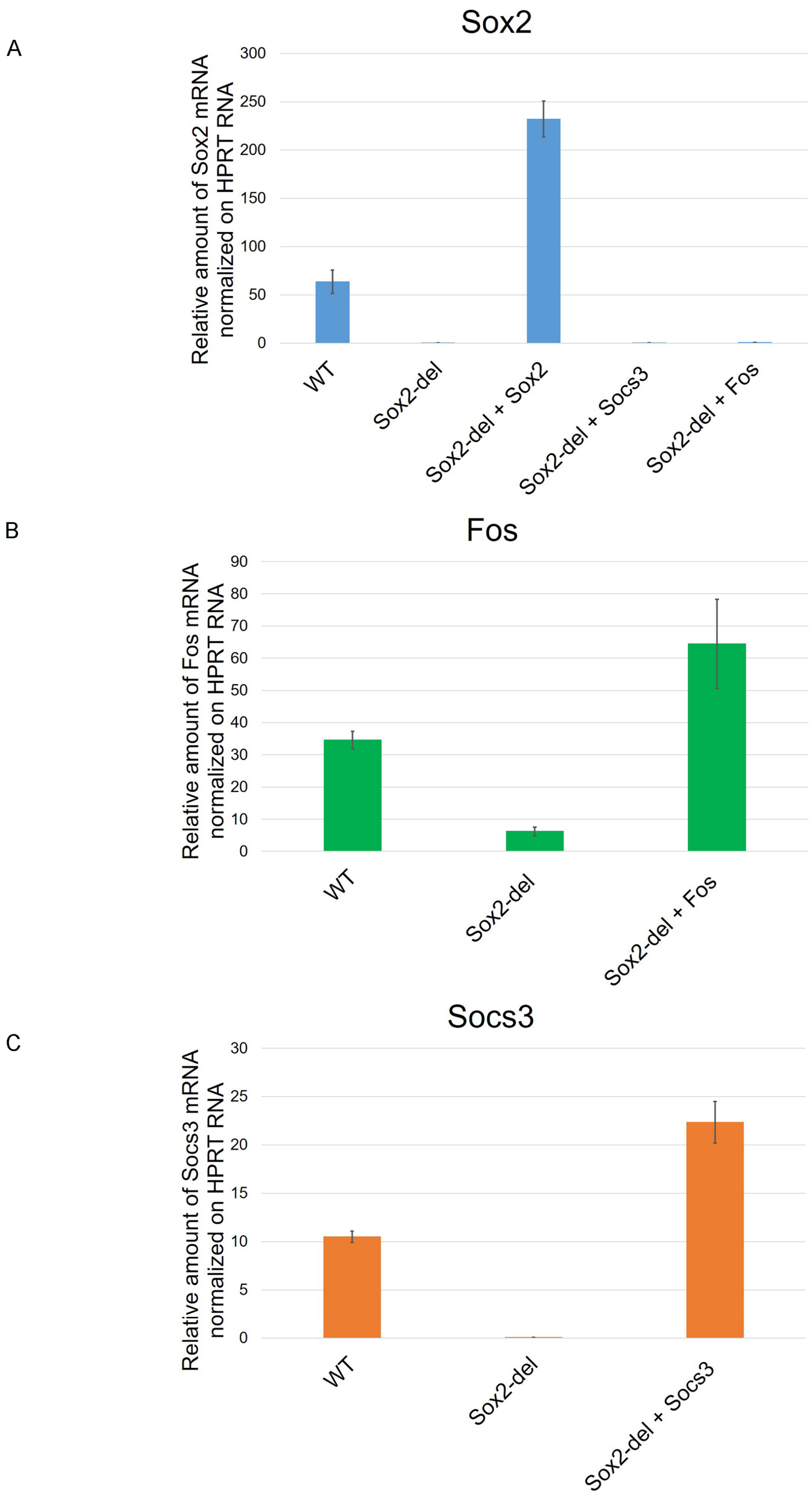

Figure S2. RT-PCR quantitation of Sox2, Fos and Socs3 mRNA in WT, Sox2-del and Sox2-del NSC transduced with Sox2, or Fos or Socs3-expressing lentiviruses. qRT-PCR was performed in triplicate.

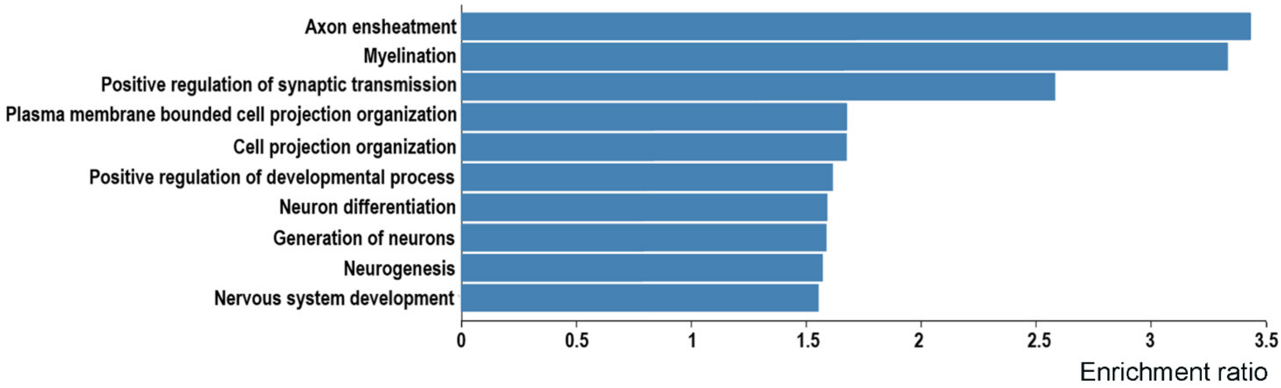

Figure S3. GO-biological process analysis for genes downregulated in undifferentiated Sox2-del NSC. The list of overall downregulated genes (TPM>5) was obtained from [8]; genes with a quality value >0.9 and whose expression is decreased more than 2 times (i.e. whose log2FC is less than -1) were considered downregulated. The 989 genes extrapolated in this way were then subjected to GO-biological term analysis on the online tool WebGestalt [15] as described in Material and Methods. Out of the 989 downregulated genes, 721 were mapped to one or more GO terms; the bar plot represents the TOP10 most enriched GO terms. Lists of genes belonging to each GO term are given in Supplementary Table 1 (sheet: enriched gene set GO analysis).

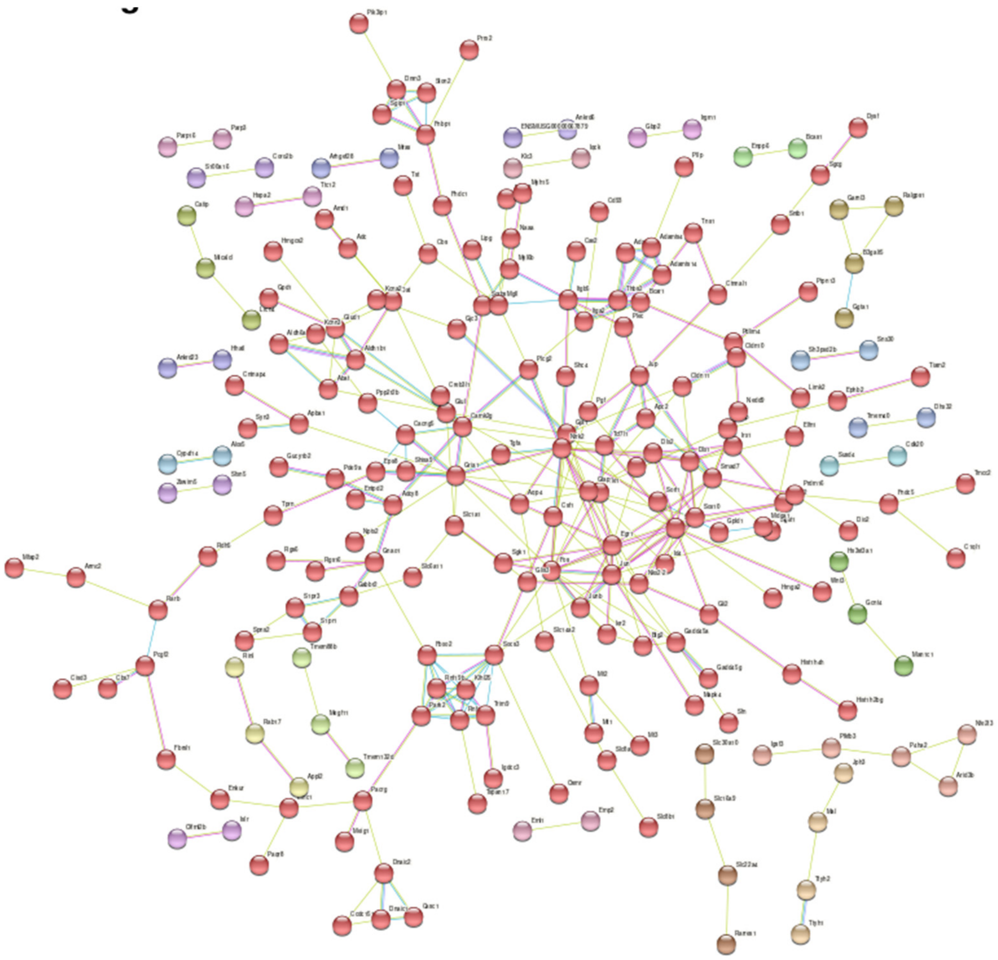

Figure S4. The whole interaction network from Fig. 6, showing the biggest network using MCL 1.1.

Table S1. RNA-Seq Expression Data for WT and Sox2-del NSC at D4 and D11.

For the corresponding D0 data, see Bertolini et al., Cell Stem Cell 2019, 24:462-476.

Table S2. Lists of genes belonging to each GO term in Fig. S2 are given (sheet: enriched gene set GO analysis). In the second sheet (DE neuronal genes, section "Downregulated in SOX2-deleted"), on the left (columns A-E, Gene and GO term), the GO term corresponding to each gene is indicated. In the right column (F, Overall neuronal genes), the list of genes appearing in one or more GO category is presented; this list corresponds to 201 genes out of the 989 downregulated genes belonging to GO terms related to neuronal differentiation, maturation, or maintenance (.xcl file).

Table S3. GREAT annotation of subnucleosomally enriched peaks shared between SOX2 and AP1 (.xcl file).

Table S4. DAVID Gene Ontology analysis of genes bound by SOX2 and AP1 that are expressed in NSC (.xcl file)

Table S5. Genes bound by SOX2 and AP1 and downregulated in SOX2-deleted NSC (.xcl file).

Table S6. STRING Gene Ontologies for genes bound by SOX2 and AP1 and downregulated in SOX2-deleted NSC (.xcl file)
